# Comparative effects of amiodarone and dronedarone treatments on cardiac function in a rabbit model

**DOI:** 10.14202/vetworld.2019.345-351

**Published:** 2019-02-28

**Authors:** Worakan Boonhoh, Anusak Kijtawornrat, Suwanakiet Sawangkoon

**Affiliations:** 1 Department of Physiology, Animal Physiology Program, Faculty of Veterinary Science, Chulalongkorn University, 39 Henri Dunant Road, Pathumwan, Bangkok 10330, Thailand; 2 Department of Physiology, Faculty of Veterinary Science, Chulalongkorn University, 39 Henri Dunant Road, Pathumwan, Bangkok 10330, Thailand; 3 Research Clusters: Research Study and Testing of Drug’s Effect Related to Cardiovascular System in Laboratory Animal, Chulalongkorn University, 39 Henri Dunant Road, Pathumwan, Bangkok 10330, Thailand

**Keywords:** Amiodarone, cardiac function, dronedarone, heart rate variability, rabbit

## Abstract

**Aim:**

The objective of the study was to compare the effects of amiodarone (AM) and dronedarone (DR) on heart rate variability (HRV) and cardiac contractility in a rabbit model.

**Materials and Methods:**

A total of 16 male New Zealand white rabbits were divided into two groups, treated either with AM or DR at incremental dosages of 50 mg/kg/day (AM50 and DR50) and 100 mg/kg/day (AM100 and DR100), orally administrated for 7 days. At the end of each period, electrocardiograms were recorded during consciousness and analyzed using the short-term time and frequency domains of HRV. Standard echocardiography and speckle-tracking echocardiography were studied during immobilization with xylazine and ketamine.

**Results:**

The results showed that AM100 and DR100 significantly decreased heart rate, total power, low-frequency component, and low-to-high frequency ratio compared with baselines. Most echocardiogram parameters revealed no significant difference from baselines, except for the global circumferential plane strain rate and time to peak standard deviation of strain, which had statistical significances after treating with AM.

**Conclusion:**

Both AM and DR possess negative chronotropy and reduce HRV, which may be explained by their sympathetic suppression and calcium channel blocking activities. Theoretically, both antiarrhythmic drugs may also possess negative inotropy, but only AM is shown to have a negative inotropic effect and reduces cardiac dyssynchrony in this model.

## Introduction

Amiodarone (AM) and dronedarone (DR) are multichannel blocking agents that were classified as Class III antiarrhythmic drugs, though they may have the potential to cover all four classes. AM contains iodine in its structure, similarly to thyroxine (T4) [1], and may have iodine up to 37% by weight [2]. This iodine-retaining structure may interfere with the actions of thyroid hormones and their synthesis. There are reports that AM can induce thyroid dysfunction either in thyrotoxicosis or hypothyroidism depending on the individual iodine status [3-6], and the daily maintenance dose of AM may exceed the daily requirement of iodine intake [7]. However, DR was synthesized without iodine in its molecule to avoid these adverse effects in patients. Theoretically, it may not produce thyroid dysfunction, and several studies have shown that DR did not alter thyroid hormone levels [8,9]. However, there has been some evidence that DR could alter thyroid hormone levels in rabbits and rats after treatment for a couple of weeks [3,10]. Thyroid hormones are known to have actions on cardiac functions, in increased heart rate (HR), contractility, and cardiac output and decreased systemic vascular resistance [7]. Therefore, AM and DR may interfere with cardiac functions, both chronotropy and inotropy, due to dysfunction in thyroid hormones, beta-adrenergic receptor (BAR) blockade, and calcium channel blockade [9,11]. Although the sympathovagal balance may have some influence, a prospective study found that patients with myocardial infarction, who benefitted from prophylactic treatment with AM, tended to have improved HR variability (HRV) [2,12]. AM injection also increased HRV in rats, wherein AM acutely raised vagal activity, while sympathetic activity was shortly increased and decreased afterward [13]. However, the training condition for data collection to analyze HRV and the use of these data for prediction in a rabbit model are still unclear. Using fast Fourier transform (FFT) and newly released software optimized for each species will facilitate the use of this technique in animal models such as rabbits.

In the past, it was a challenge to evaluate the cardiac contractility in small animals using a non-invasive technique such as echocardiography due to the difficulty of the technique and limitations of technology. However, the emergence of new digital imaging technology during this decade will make it possible. Speckle-tracking echocardiography (STE), which is used to calculate strain (St) and strain rate (SR), may be employed to evaluate cardiac contractility with less load dependence than standard echocardiography (S-Echo) [14-16]. Until now, there has been a limitation on the data available for HRV and echocardiography in the rabbit model, together with a discrepancy in the drug action, especially AM and DR in this model.

Therefore, we aimed to study the effects of AM and DR treatments on HRV and cardiac contractility using FFT and the echocardiographic technique in a rabbit model.

## Materials and Methods

### Ethical approval

The present study was approved by the Institutional Animal Care and Use Committee of Chulalongkorn University, Bangkok, Thailand. The use of animals had been followed guidelines in the Guide for the Care and Use of Laboratory Animals, by the US National Research Council of the National Academies.

### Animal experimentation

A total of 16 male New Zealand white rabbits weighing between 1.6 and 2.5 kg were randomly divided into two groups: AM (n=8) and DR (n=8). Two animals were trained to stay in restrainers together for 1 h daily during the 1^st^ week. Then, the animals were orally administered AM or DR diluted in 2.5 mL propylene glycol at a dosage of 50 mg/kg daily (AM50 and DR50) in the 2^nd^ week and a dosage of 100 mg/kg daily (AM100 and DR100) in the 3^rd^ week.

**Figure-1:**
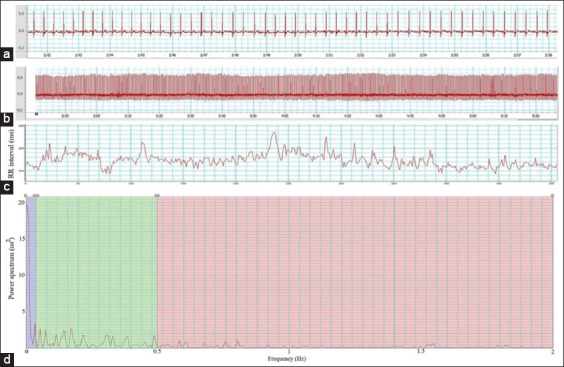
The heart rate variability; editing procedure a - Rabbit electrocardiogram (ECG), b - ECG 512 beats, c - Tachogram, and d: Power spectrum plot.

Electrocardiogram (ECG) and echocardiogram readings were collected for baseline values on a day before treatment started and on the last day of each treatment period. ECGs of conscious rabbits were recorded using a commercial setup (PowerLab, ADInstruments, New Zealand) while the rabbits were in the restrainer. HR, time domain, and frequency domain of HRV were calculated from 512 consecutive normal RR (NN) intervals using the LabChart program (Figure-1). Standard deviation of all NN intervals (SDNN), percentage of pairs of adjacent NN intervals differing by >20 ms (pNN20), percentage of pairs of adjacent NN intervals differing by >10 ms (pNN10), and the square root of the mean of the sum of the squares of differences between adjacent NN intervals (RMSSD) were used to represent changes in the time domain. The program computed the FFT algorithm, and the criteria were configured for the rabbit species. The spectral bands, such as very low frequency (VLF), low frequency (LF), and high frequency (HF), were set at 0.0-0.04, 0.04-0.5, and 0.5-2.0 Hz, respectively.

**Figure-2:**
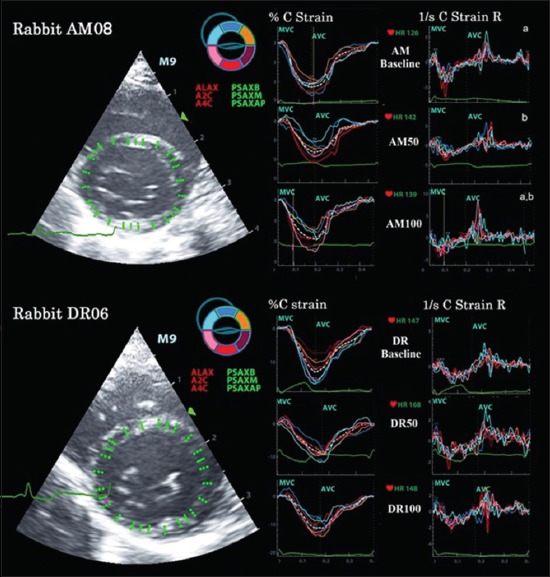
Speckle-tracking echocardiograms of global strain and strain rate of circumferential planes at the basal segmental level, obtained from the right parasternal short axis view of the anesthetized rabbits received AM and DR. AM - Amiodarone, AVC - Aortic valve closure, C strain - Circumferential strain, C Strain R - Circumferential strain rate, DR - Dronedarone, MVC - Mitral valve closure.

Echocardiograms were used to evaluate the cardiac contractility after immobilizing the rabbit with xylazine (4 mg/kg IM) and ketamine (17 mg/kg IM). Animals were placed in right lateral recumbency on an echocardiographic table that had a proper hole for placing the ultrasound probe from beneath. ECG flat-jaw electrode clips were clamped on the animals’ limbs. Five consecutive beat echocardiograms were collected using a commercial ultrasound system with a 4-10 MHz phased array probe (Mindray M9, Mindray, China). The S-Echo parameters of the left ventricle were obtained from the right parasternal short-axis views for the calculation of ejection fraction (EF), fractional shortening (FS), pre-ejection period (PEP), and ejection time (ET) and the left apical five-chamber view for the calculations of isovolumic contraction time (IVCT), isovolumic relaxation time (IVRT), and Tei index. STE readings were obtained from the right parasternal short axis of the left ventricle for the basal segment (at the mitral valve leaflets), middle segment (at the papillary muscle), and apical segment (at the level closed to the apex) for calculating St and SR of the radial and circumferential planes (Figure-2). The STE data were analyzed using an offline tissue tracking software package (Tissue Tracking QA, Mindray, China). The cardiac movement was manually marked along the interventricular septum and the left ventricular free wall, and the program automatically computed global St and SR of the radial and circumferential planes at basal, middle, and apical segments and time to peak standard deviation (TPSD).

### Statistical analysis

All data were shown in mean±standard error. Data in the same group were analyzed using one-way ANOVA repeated measures design, followed by the Student–Newman–Keuls *post hoc* test. To compare cardiac effects between AM and DR treatments, the data between two groups at the same dosage were analyzed using Student’s t-test. A p<0.05 was considered to be statistically significant.

## Results

HR, obtained during a fully conscious state, decreased after treatment with AM and DR, especially at the dose of 100 mg/kg. There was no significant difference in HR between AM and DR treatments. SDNN, pNN20, pNN10, and RMSSD in the time domain of HRV did not change significantly after treatments. However, both treatments significantly affected some parameters of the frequency domain of HRV. Total power (TP), LF, and LF/HF ratio were decreased in a dose-dependent manner after treating with both AM and DR. However, neither compound seemed to have significant effects on VLF and HF components (Table-1).

**Table-1:** Heart rate and heart rate variability.

Index	AM treatment (n=8)			DR treatment (n=8)		
	
	Baseline (Day 0)	AM50 (Day 7)	AM100 (Day 14)	Baseline (Day 0)	DR50 (Day 7)	DR100 (Day 14)
HR (bpm)	218.61^a^±8.40	199.69^a,b^±14.49	192.21^b^±7.12	207.86^a^±5.69	204.81^a^±6.37	188.33^b^±7.23
SDNN (ms)	14.34±2.37	11.96±0.88	11.77±1.23	14.54±0.98	12.71±1.28	11.37±1.49
pNN20 (%)	2.62±1.23	1.81±0.72	2.93±1.13	3.15±1.02	4.45±2.20	2.42±0.98
pNN10 (%)	14.28±4.98	12.87±3.74	17.03±4.44	19.63±3.88	21.05±5.52	15.31±4.80
RMSSD (ms)	7.22±1.14	6.87±0.77	7.70±1.11	8.64±0.95	9.30±1.31	7.34±1.03
TP (ms^2^)	193.07^a^±23.95	166.24^ab^±17.64	139.37^b^±18.57	216.08^a^±23.08	182.13^ab^±33.90	137.43^b^±31.41
VLF (ms^2^)	92.15±18.72	59.83±13.77	58.86±12.19	99.89±18.38	79.20±19.50	71.91±17.48
LF (ms^2^)	86.89^a^±11.85	78.88^a^±10.08	52.46^b^±6.19	92.26^a^±11.32	71.30^a^±15.33	37.03^b^±8.18
HF (ms^2^)	19.38±4.03	30.36±9.29	30.85±5.20	31.32±7.48	41.06±12.41	30.83±8.56
LF/HF ratio	5.39^a^±0.89	3.99^ab^±0.83	2.32^b^±0.65	3.80^a^±0.64	2.28^b^±0.54	1.54^b^±0.24

Different letters ^a,b^within the same drug treatments represent significant differences (p<0.05). HF=High-frequency band, HR=Heart rate, LF=Low-frequency band, pNN10=Percentage of pairs of adjacent NN intervals differing by>10 ms, pNN20=Percentage of pairs of adjacent NN intervals differing by>20 ms, RMSSD=The square root of the mean of the sum of the squares of differences between adjacent NN intervals, SDNN=Standard deviation of all NN intervals, TP=Total power, VLF=Very low-frequency band, AM=Amiodarone, DR=Dronedarone

Neither treatment significantly changed S-Echo parameters such as EF, FS, PEP/ET, IVCT, IVRT, and Tei index (Table-2). We observed some significant changes using the STE technique to estimate the St and SR from the radial and circumferential movement of the left ventricle. In the radial plane of the left ventricle, the global St and SR of the basal, middle, and apical segments did not reach any significant levels after treating with both AM and DR (Table-3). However, the global SR of the circumferential plane tended to decrease after treating with both AM and DR, and statistical significance (p<0.05) was observed at the average of all segments after treating with AM (Table-4). TPSD of the circumferential St also decreased after treating with AM, especially at the dose of 100 mg/kg (Table-4).

**Table-2:** Standard echocardiography.

Index	AM treatment (n=8)			DR treatment (n=8)		
	
	Baseline (Day 0)	AM50 (Day 7)	AM100 (Day 14)	Baseline (Day 0)	DR50 (Day 7)	DR100 (Day 14)
EF (%)	64.98±2.78	64.88±2.76	61.53±3.50	60.22±0.86	58.61±1.51	60.50±3.20
FS(%)	32.73±2.03	32.71±1.76	30.77±2.35	29.27±0.50	30.14±1.83	29.95±2.11
PEP/ET	0.19±0.01	0.19±0.01	0.17±0.01	0.22±0.01	0.19±0.01	0.18±0.01
IVCT (ms)	48.75±2.97	45.13±2.02	48.38±1.88	48.79±2.27	49.63±2.43	47.08±2.09
IVRT (ms)	49.67±1.09	48.67±1.95	48.92±1.44	47.42±1.60	50.29±2.06	50.04±2.16
Tei index	0.76±0.04	0.72±0.04	0.73±0.02	0.75±0.02	0.73±0.03	0.70±0.01

EF=Ejection fraction, FS=Fractional shortening, IVCT=Isovolumic contraction time, IVRT=Isovolumic relaxation time, PEP=Pre-ejection period, ET=Ejection time, AM=Amiodarone, DR=Dronedarone

**Table-3:** Global strain and strain rate of radial planes at basal, middle, and apical segments.

Index	AM treatment (n=8)			DR treatment (n=8)		
	
	Baseline (Day 0)	AM50 (Day 7)	AM100 (Day 14)	Baseline (Day 0)	DR50 (Day 7)	DR100 (Day 14)
Basal segment						
St (%)	2.25±0.92	9.30±5.23	8.90±3.89	3.55±1.78	2.70±0.85	7.88±1.99
SR (1/s)	2.00±0.61	3.27±0.99	2.36±0.72	2.39±0.22	1.78±0.22	2.50±0.14
Middle segment						
St (%)	10.43±2.09	11.49±1.77	11.46±2.22	14.36±1.96	15.32±3.25	8.05±1.81
SR (1/s)	2.41±0.24	2.65±0.24	2.54±0.23	2.71±0.21	2.92±0.48	2.48±0.45
Apical segment						
St (%)	12.93±2.69	9.60±2.78	11.83±3.26	13.5±2.68	8.25±1.61	13.23±1.97
SR (1/s)	2.80±0.39	2.33±0.27	2.25±0.50	2.34±0.38	1.93±0.17	2.52±0.39
Average of all segments						
St (%)	7.90±1.06	10.13±1.96	10.73±2.39	10.47±1.17	8.75±1.15	9.51±1.28
SR (1/s)	2.40±0.23	2.74±0.34	2.81±0.53	2.48±0.19	2.21±0.20	2.49±0.22
TPSD						
St (ms)	49.83±4.78	57.23±4.20	47.58±5.53	45.52±5.27	50.44±1.91	48.89±4.36
SR (ms)	31.92±2.88	36.93±4.38	37.08±3.13	35.95±3.99	38.25±4.16	38.34±2.84

SR=Strain rate, St=Strain, TPSD=Time to peak standard deviation, AM=Amiodarone, DR=Dronedarone

**Table-4:** Global strain and strain rate of circumferential planes at basal, middle, and apical segments.

Index	AM treatment (n=8)			DR treatment (n=8)		
	
	Baseline (Day 0)	AM50 (Day 7)	AM100 (Day 14)	Baseline (Day 0)	DR50 (Day 7)	DR100 (Day 14)
Basal segment						
St (%)	−13.81±0.83	−11.63±1.37	−13.58±0.99	−11.24±1.28	−10.63±1.14	−11.55±0.84
SR (1/s)	0.46±0.12	0.24±0.08	0.26±0.06	0.18±0.06	0.22±0.05	0.14±0.04
Middle segment						
St (%)	−11.14±1.02	−10.52±1.45	−9.79±0.31	−7.88±0.85	−7.76±0.58	−9.55±0.72
SR (1/s)	0.33±0.08	0.27±0.05	0.16±0.04	0.27±0.08	0.21±0.04	0.20±0.08
Apical segment						
St (%)	−8.55±0.97	−8.31±0.55	−8.61±0.52	−6.61±0.93	−6.82±1.29	−8.96±1.16
SR (1/s)	0.37±0.09	0.25±0.06	0.20±0.06	0.15±0.04	0.32±0.08	0.15±0.05
Average of all segments						
St (%)	−10.71±0.92	−10.15±0.63	−10.66±0.40	−8.70±0.93	−8.28±0.61	−10.02±0.60
SR (1/s)	0.39^a^±0.09	0.25^b^±0.03	0.21^b^±0.03	0.20±0.03	0.25±0.03	0.17±0.04
TPSD						
St (ms)	23.95^a^±3.77	22.88^a^±2.83	15.09^b^±1.74	20.16±2.99	19.25±3.93	18.49±3.37
SR (ms)	48.30±7.37	59.76±7.66	57.65±5.71	57.81±4.14	58.60±2.50	59.67±5.90

Different letters ^a,b^within the same drug treatments represent significant differences (p<0.05). SR=Strain rate, St=Strain, TPSD=Time to peak standard deviation, AM=Amiodarone, DR=Dronedarone

## Discussion

In general, the HR and rhythm are influenced by the autonomic nervous system, which plays the roles on top of the intrinsic HR generated from the sinoatrial (SA) node. AM and DR possess both beta-adrenergic blocking and calcium channel blocking activities that may interfere with the heart function. In the present study, rabbits after receiving AM and DR showed a negative chronotropic effect, especially at the dose of 100 mg/kg. In these cases, the beta-blocking activity may reduce sympathetic activity and enhance parasympathetic activity. Furthermore, the calcium channel blocking activity may also reduce the calcium influx during the depolarization phase of the SA and atrioventricular node. Thus, the marked negative chronotropy was shown after the administration of AM and DR.

Over the past few decades, HRV has been broadly used to monitor the effect of autonomic control on the heart. It could predict mortality risk in patients with cardiovascular and non-cardiovascular diseases [17]. The LF component of HRV represents the sympathovagal activity, and the vagal system influences the HF component system [18,19]. Nevertheless, breathing variability may also involve the LF component [20]. In the present study, we performed short-term analyses of both time and frequency domains of HRV in rabbits, and we found that TP was decreased in both the AM- and DR-treated groups. This was mainly due to the decrease of LF components in a dose-dependent manner. This effect is similar to some studies in rats, wherein AM increased vagal activity and decreased sympathetic activity, causing a significant reduction in LF [8,21]. This may emphasize the effect of both drugs on beta-adrenergic inhibition, leading to sympathetic suppression in this rabbit model, the same as in the rat model. In addition, both treatments also reduced the LF/HF ratio due to a decrease in the LF component but not the HF component. These findings potentially suggest that these drugs may not directly affect the parasympathetic system of the rabbit even though the parasympathetic activity may be theoretically enhanced due to sympathetic activity suppression. Finally, the VLF component was unchanged in both the treated groups. Normally, there is an association of the VLF with thermoregulation and plasma renin activity. However, a short term of HRV with 512 samples may not be enough to provide information, or these drugs may not have any influence on the thermoregulation and renin activity in these normal rabbits.

To understand the effects of these antiarrhythmic drugs on inotropy of the rabbit heart using a non-invasive technique, we performed S-Echo and STE to determine cardiac contractility in the rabbit model. We found all parameters related to S-Echo; including EF, FS, PEP/ET, IVCT, IVRT, and Tei index, remained unchanged in both AM and DR treatments compared to baselines. We know theoretically that both beta-adrenergic blocking property and calcium channel blocking activity of AM and DR can suppress cardiac contractility from decreased calcium influx that initiates the calcium-induced calcium release process. However, none of the S-Echo parameters reflected the negative inotropy in this model. This may be explained by the fact that these drugs slow the HR and may cause an increase of preload that, in turn, increases cardiac contractility from the Frank–Starling mechanism in this *in vivo* model. Furthermore, previous studies showed that DR reduced the HR without affecting cardiac contractility in anesthetized pigs [22], and short-term oral DR treatment may have no effect on cardiac inotropy and dromotropy in conscious telemetric dogs [23]. However, there were studies, suggesting that AM and DR could decrease tension of the isolated ventricular muscle in guinea pigs [24,25]. This *in vitro* model, which has no preload variation, demonstrated the negative inotropy of DR. Therefore, this discrepancy may need other approaches to demonstrate the negative inotropy in cases of *in vivo* studies to minimize the pre-load effect. In comparison with S-Echo, many previous studies have found that STE may be more sensitive than S-Echo for demonstrating cardiac contractility [14-16], and STE detection covers most of the LV segments. In the present study, we found AM decreased SR of STE, and statistical significance of the circumferential SR of the average of all segments was observed in doses of 50 mg/kg and 100 mg/kg; this may be a better example for representing the negative inotropy of AM. One report studied the effect of unloading on St and SR in a porcine model. The researchers found that SR has lesser load-dependent effect compared with St, and either radial SR or circumferential SR also has a highly significant correlation with dP/dt_max_ [26]. The porcine model, together with the rabbit model in our study, has shown that SR is a good predictor of cardiac contractility. Moreover, only AM at the dose of 100 mg/kg could significantly reduce the TPSD of the circumferential St. This result potentially suggests that AM may reduce cardiac dyssynchrony more than DR does, and TPSD may be used as an index for the assessment of the left ventricular dyssynchrony in this model the same as a result found in a human study [27].

## Conclusion

AM and DR possess negative chronotropy and decrease TP and the LF component of HRV in a rabbit model, suggesting that they may inhibit BAR in the heart, leading to sympathetic suppression. AM and DR have minimal negative inotropy. Only the SR of STE can demonstrate the negative inotropy of AM in this model. Moreover, AM may also reduce the cardiac dyssynchrony more than DR does, as demonstrated from a decrease of the circumferential TPSD. All these results suggest that rabbits can be used as models for studying the effects of Class III antiarrhythmic drugs on HRV and cardiac function.

## Authors’ Contributions

WB was responsible for animal care and use and data collection. AK was involved in experimental technique and data reviews. SS was responsible for conducting experimental design, statistical analysis, and experimental technique for both HRV and echocardiographic studies. All authors read and approved the final manuscript
